# Platelet Count Response to *Helicobacter pylori* Eradication in Iranian Patients with Idiopathic Thrombocytopenic Purpura

**DOI:** 10.4084/MJHID.2012.056

**Published:** 2012-08-10

**Authors:** Mehrdad Payandeh, Nasrollah Sohrabi, Mohammad Erfan Zare, Atefeh Nasir Kansestani, Amir Hossein Hashemian

**Affiliations:** 1 Department of Medical Laboratory Sciences, Paramedicine Faculty, Kermanshah University of Medical Sciences, Kermanshah, Iran.; 2 Medical Biology Research Center, Kermanshah University of Medical Sciences, Kermanshah, Iran.; 3 Student Research Committee, Kermanshah University of Medical Sciences, Kermanshah, Iran.; 4 Department of Biostatistics, Faculty of Public Health, Kermanshah University of Medical Sciences, Kermanshah, Iran.

## Abstract

Idiopathic thrombocytopenic purpura (ITP) is an autoimmune hematological disorder characterized by auto antibody-mediated platelet destruction. Although the main cause of ITP remains unclear, but its relationship with some infection was demonstrated. In recent years, many studies have demonstrated improvement of platelet counts in ITP patients after treating *Helicobacter pylori* infection. The aim of this study was to investigate the effects of *H. pylori* eradication on platelet count response in Iranian ITP patients.

A total of 26 patients diagnosed with both ITP and *H. pylori* infection. ITP were diagnosed whose platelet counts were less than 100×10^3^/μL. These patients were tested for *H. pylori* infection by Urea Breath Test and serum *H. pylori* antibody. All patients received triple therapy for 7 or 14 days to eradicate *H. pylori* infection. These patients followed for six months.

Prevalence of *H. pylori* was 67.3%. *H. pylori* eradication achieved in 89.5% (26/29). Of the 26 patients, 15 (57.7%) exhibited a complete response (CR) and 11 (42.3%) were unresponsive. We did not find partial responders. There was a significant difference in the baseline platelet count of responders and non-responders patients (*p*<0.001). All responders had platelet count ≥50×10^3^/μL and all non-responders had platelet count <50×10^3^/μL.

Results of this study revealed that eradication therapy of *H. pylori* infection can improve platelet counts in ITP patients especially with mild thrombocytopenia and support routine detection and treatment of *H. pylori* infection in ITP patients in populations with a high prevalence of this infection.

## Introduction

*Helicobacter pylori* (*H.pylori*) is the most common chronic pathogen that colonizes the human gastric mucosa. It has been recognized as the causative agent of chronic gastritis, gastro duodenal ulcers, adenocarcinoma and mucosa-associated lymphoid tissue lymphoma (MALT).^1^,^2^ The prevalence of *H.pylori* infection in geographic regions of the world is different.^2^ This rate in the most of the Asian countries such as Japan, South Korea and Iran is too high, but in Western countries is much lower.^3^–^6^ In recent years, several studies have proposed that *H.pylori* infection may be associated with some extra gastric disease especially hematological disorders such as iron deficiency anemia, pernicious anemia and idiopathic thrombocytopenic purpura (ITP).^7^–^9^

ITP is an autoimmune hematological disorder characterized by auto antibody-mediated platelet destruction. Although the main cause of ITP remains unclear, but its relationship with some infection was demonstrated.^10^–^12^ In 1998, Gassbarrini, for first time, observed increased platelet counts after *H.pylori* eradication in ITP patients.^9^ In recent years, many studies have demonstrated improvement of platelet counts in ITP patients after treating *H.pylori* infection.^13^–^18^ But in some studies, were observed no favorable effect on patients with ITP.^19^,^20^ The discrepancy might be due to different strains of *H.pylori* in these geographic regions. In this study we investigated the effects of *H.pylori* eradication on platelet counts in ITP patients in a teaching hospital in Kermanshah, west of Iran.

## Material and Methods

In this retrospective study, between June 2009 and November 2010, 52 patients with ITP who attended the Taleghani hospital in Kermanshah, west of Iran, were evaluated. ITP was diagnosed according to the standard criteria and defined by thrombocytopenia (platelet counts ≤100×10^3^/.^21^ Other causes of thrombocytopenia such as thrombocytopenia caused by drugs, pseudothrombocytopenia, hepatitis C virus infection, hepatitis B virus infection, human immunodeficiency virus infection and autoimmune disorders were excluded. The patients also were excluded if they had been received eradication therapy for *H.pylori* infection within 2 years or an antibiotic or proton pump inhibitor within the previous 4 weeks.

We used Urea Breath Test (UBT) and serum *H.pylori* antibody for diagnosis of *H.pylori* infection.^22^ All patients with *H.pylori* infection was treated with standard eradication therapy included amoxicillin 1000 mg twice daily, clarithromcin 500 mg twice daily, and a proton pump inhibitor 40 mg twice daily for 2 weeks.^23^ Eradication of *H.pylori* was evaluated two weeks after treating by the same tests which we used for diagnosis of *H.pylori* infection. Platelet counts were monitored every 2 weeks for the first 2 months, every month for the next 4 months after the end of treatment. Complete response (CR) was defined as a platelet count ≥100×10^3^/μL at month 6. Partial response (PR) was defined by a platelet increase of ≥30×10^3^/μL and at least a doubling of the base line count at month 6. No response (NR) was defined a platelet count <30×10^3^/μL or a count increase less than 2-times the baseline count after month 6.^21^ According to declaration of Helsinki; we took consent from all patients before *H.pylori* eradication for remedy of their ITP disorders.

Differences of platelet count are expressed as the mean (SD) as appropriate. An ANOVA test was used for analysis of platelet differences in 3 groups (CR, PR and NR); the t-test was used to compare positive and negative response. A P-value of less than 0.05 was considered statistically significant. All statistical analysis were performed by using SPSS software version 16.0.

## Results

**Figure 1:**
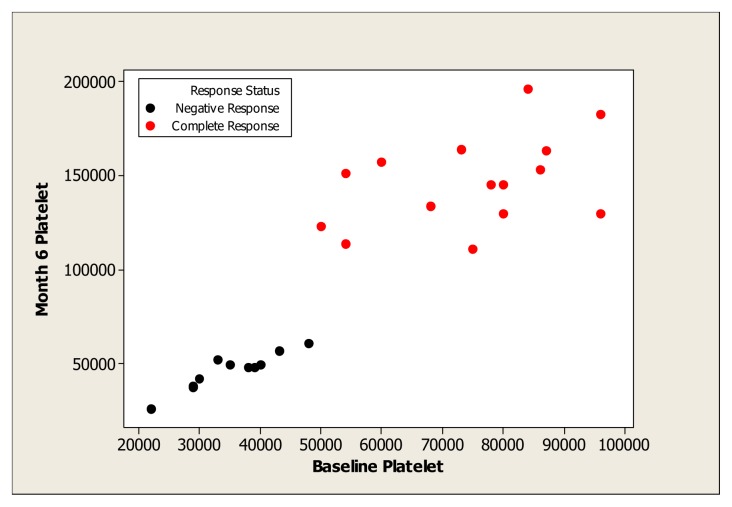
Platelet count response status after 6 months vs. Baseline Platelet. CR: Complete Response; NR: No Response

Of 52 patients with ITP, *H.pylori* infection was found in 67.3% (35/52) of patients. Three patients with autoimmune disorders, two patients with HBV infection and one patient with HCV infection were excluded. Thus 29 patients were considered whom 26 (13 males, 13 females, mean age 38.2 years) achieved *H.pylori* eradication (89.6%). After *H pylori* eradication, CR was obtained in 57.7% (15/26) of patients (CR= platelet count ≥100×10^3^/μL); 11 patients (42.3) were unresponsive. No PR was found. (Table 1). There is a significant difference between the platelet counts of responders and non-responders (*p*<0.001) (Table 2). All responders had platelet count ≥50×10^3^/μL and all non-responders had platelet count <50×10^3^/μL (Table 1 and Figure 1).

## Discussion

ITP is an autoimmune blood disorder in which platelet destruction is mediated by anti-platelet antibodies.^7^–^9^ The mechanisms of anti-platelet antibodies development are still a little known. The association between ITP and some infections has been reported previously.^10^–^12^ For first time, Gasbarrini in 1998 proposed that *H.pylori* infection may be associated with ITP.^9^ In recent years, many studies have demonstrated improvement of platelet counts in ITP patients after treatment of *H.pylori* infection.^13^–^18^

In our study, the prevalence of *H.pylori* infection in ITP patients was 69.3%. This rate similar to prevalence of *H.pylori* infection in the general population in Iran.^5^ These results also were comparable to those of previous studies which were done in Asian countries.^6^ such as Japan and South Korea but this rate is in contrast studies were conducted in Western countries.^3-^ In general, the prevalence of *H.pylori* infection varies according to geographic location and in Asian countries such as Iran is too high.^3^–^5^

In this study all patients with *H.pylori* infection were treated with triple therapy regimen and eradication rate was 86.5%. This finding is in agreement to other studies which have showed successful eradication greater than 70% using triple therapy.^17^,^18^

In this study 65.6% of ITP patients had an increase of platelet counts after eradication of *H.pylori* infection. We found that there is a significantly association between overall response rate and eradication therapy infections (*p*<0.001). According our data in other studies, conducted by Emilia *et al,* Fujimara *et al* and Inaba *et al* respectively 68%, 63% and 44% of ITP patients showed significant increase in platelet count after *H.pylori* eradication.^16^–^18^ In contrast the effects of eradication therapy had no favorable effect on platelet count in other series. Ahn *et al* reported increased platelet count only in 7% of treated patients^19^, no platelet response were observed in ITP patients after eradication therapy of H.pylori infection in studies done by Micheal *et al* and Stasi *et al*.^20^,^25^ The discrepancy among these studies might be due to geographic variation in expression of some proteins such as Cag A (Cytotoxin-associated gene A) in different *H.pylori* genotypes.^13^,^14^ The prevalence of Cag A-positive *H.pylori* strains is different in geographical regions of the world. In Asian countries such as Japan, South Korea and Iran, most of *H.pylori* strains express Cag A;^26^–^28^ whereas the frequency of Cag A positive *H.pylori* strain in Western countries is lower.^29^ Franceschi *et al* and Takahashi *et al,* documented association between *H.pylori* eradication with disappearance of anti Cag A antibodies and significant increase in platelet counts in ITP patients; they attributed the effect of HP eradication on platelet increase to HP Cag A molecular mimicry to platelet antigens. 13,14

Thus, difference in the *H.pylori* genotypes and prevalence of Cag A positive *H.pylori* strains may explain variability in improvement of platelet counts after treatment in studies that were done in different geographic areas, but more work is needed to evaluate it formally.

In this study, all responders had platelet count ≥50×10^3^/μL and we observed poor response to treatment in ITP patients with severe thrombocytopenia. Accordingly an other study, done by Stasi *et al*, 32% of patients with mild thrombocytopenia had a platelet response, but platelet response was observed only in one patient with severe thrombocytopenia.^20^ The reason of this situation has not been addressed in most reports, but these results show that the chance of obtaining a response by HP treatment is lower in patients with severe thrombocytopenia.

## Conclusions

Results of this study revealed eradication therapy of *H.pylori* infection can improve platelet counts in ITP patients especially with mild thrombocytopenia. Also, our results show that *H.pylori* eradication cannot have a major role in the treatment of severe ITP patients. On the other hand; treating of *H.pylori* infection compared to conventional ITP treatment has some advantages such as the low cost, the non-invasiveness of diagnostic methods and favorable toxicity of drugs. Thus, this study supports routine detection and eradication of *H.pylori* infection in ITP patients in populations with a high prevalence of this infection such as Iran.

## Figures and Tables

**Table 1:** Clinical and platelet response characteristics by Patient

*Patient*			*Baseline platelet count×10^3^/μL*	*Mount 6 platelet count×10^3^/μL*	*Response status*

No	Sex	Age			
1	M	29	60	157	CR
2	F	28	84	196	CR
3	M	34	73	164	CR
4	F	23	86	153	CR
5	M	57	54	151	CR
6	M	37	87	163	CR
7	F	32	96	183	CR
8	M	17	78	145	CR
9	F	48	54	114	CR
10	M	38	80	145	CR
11	F	19	75	111	CR
12	F	32	96	130	CR
13	F	42	80	130	CR
14	F	27	50	123	CR
15	M	43	68	134	CR
16	M	54	43	57	NR
17	M	43	29	38	NR
18	M	37	22	26	NR
19	M	33	35	49	NR
20	F	33	38	48	NR
21	F	27	30	42	NR
22	F	65	33	52	NR
23	M	71	40	49	NR
24	M	19	48	61	NR
25	F	38	29	37	NR
26	F	67	39	48	NR

**Table 2:** Differences of platelet according to outcome groups

Outcome	N (%)	Median	Minimum	Maximum	Mean SD	P-Value
**No response**	11 (42.3)	10000	4000	19000	11000+/− 3974.9	
**Complete response**	15 (57.3)	67000	34000	112000	79917+/− 20042.9	< 0.001
**Total**	26 (100)	43000	4000	112000	69000 21851.8	
